# Design of an affordable IoT open-source robot arm for online teaching of robotics courses during the pandemic contingency

**DOI:** 10.1016/j.ohx.2020.e00158

**Published:** 2020-11-13

**Authors:** Victor H. Benitez, Rodrigo Symonds, David E. Elguezabal

**Affiliations:** a Department of Industrial Engineering, Universidad de Sonora, Hermosillo 83000, Mexico; b Tecnologico de Monterrey, School of Engineering and Sciences, Blvd. Enrique Mazón López 965, Hermosillo 83000, Sonora, Mexico

**Keywords:** Robot kinematics, Teaching robotics, Virtual laboratory, Online teaching, Educational innovation, Higher education

## Abstract

This article explains the design and construction of an affordable, open-source robot arm for online teaching of robotics courses. The main goal of the proposed robotic prototype is to deal with the current situation of pandemic contingency, where students and instructors cannot attend laboratory facilities in person. The robotic system has four main components: an electromechanical robot arm structure, a control system, a Wi-Fi communications module, and a human-machine interface. The IoT (Internet of Things) robot arm can be used to demonstrate important robotics topics such as direct and inverse kinematics, which are shown by programming simple and complex motions using the Denavit-Hartenberg (DH) methodology. The capabilities of the robotic system are empowered by IoT technology, which is demonstrated with an HMI interface deployed in a smartphone using wireless Wi-Fi communication through an ESP32 microcontroller. The arm's purpose is to be a low-cost and replicable robot that aids the comprehension of robotics design through project-based learning, from the theoretical aspects to the actual coding and construction of a prototype.

## Highlights

• The robot arm can be used to teach online robotics courses during covid-19 outbreak.
• The robot can be teleoperated via online app.
• Topics such as motion control, trajectory tracking, and kinematics are considered.

## Graphical abstract

**Figure:**
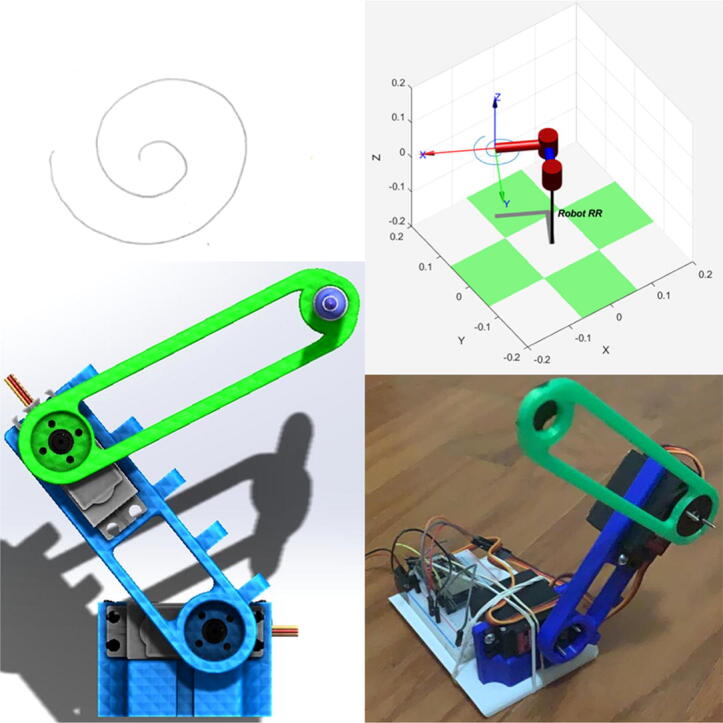

**Specifications table**

**Table:** 

Hardware name	Two-link Robot Arm
Subject area	● Educational Tools and Open-Source Alternatives to Existing Infrastructure
Hardware type	● Electrical engineering and computer science ● Mechanical engineering and materials science
Open Source License	GNU General Public License (GPL)
Cost of Hardware	$28.48 USD
Source File Repository	https://doi.org/10.17605/OSF.IO/EN9W6

## 1 Hardware in context

The COVID-19 has been declared a worldwide pandemic. This outbreak has had enormous consequences for educational institutions and raises questions regarding safety in laboratory facilities and the corresponding laboratory practices carried out there by students and research personnel. The situation is riskier or presents a higher risk of disease contagion for engineering students who perform experimental trials that require equipment and collaborative work. Most educational institutions have reduced the exposure by delivering virtual distance education to reduce the risk of contracting the virus. However, the experimentation required by laboratory practices is very difficult or impossible to achieve in a virtual environment. This has caused teaching to be seriously compromised when theory cannot be linked to practice. Some scholastic subjects that are practical or theoretical-practical have been suspended due to the confinement caused by the social distancing measures implemented by governments and health organizations to diminish the global prevalence of COVID-19.

**Fig. 1:**
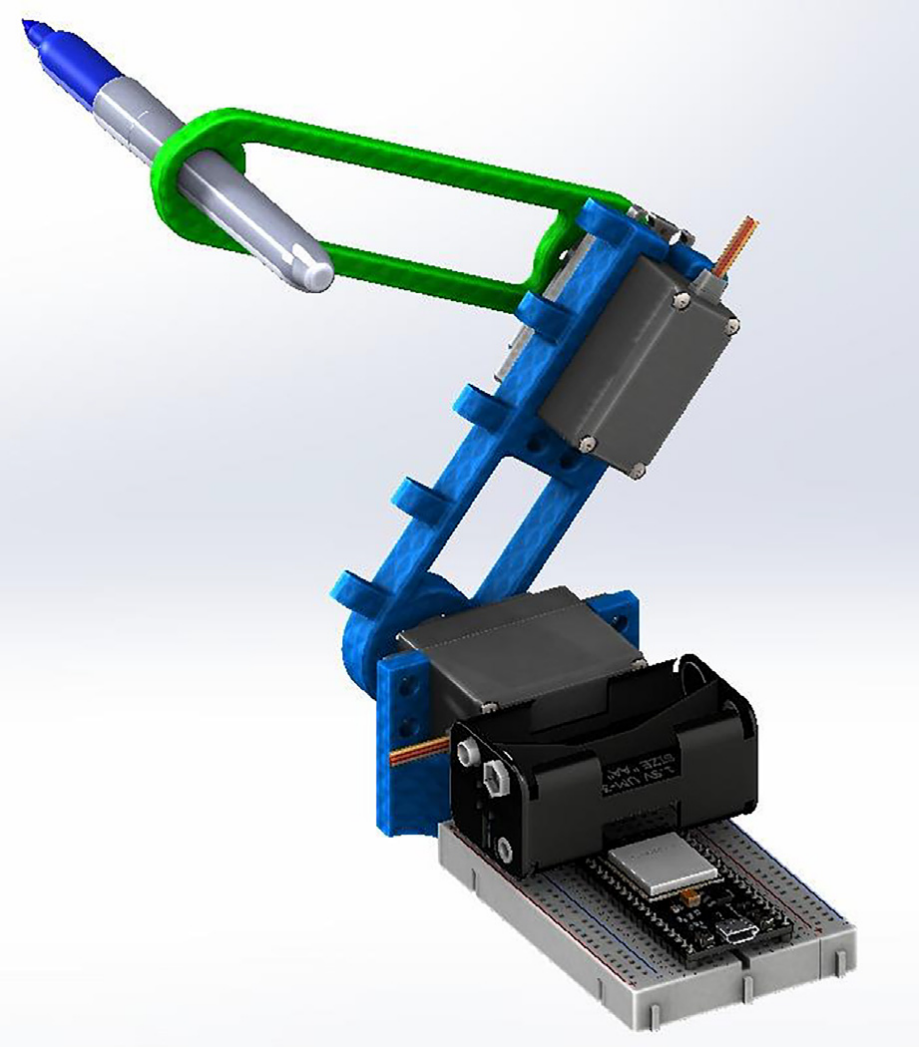
CAD rendering of the robot in SolidWorks.

The Robot Arm developed is a low – cost, robotic-arm-prototype designed to demonstrate the applications of direct and inverse kinematics using cheap and commercially available components. The motion control is achieved via an IoT interface integrated with low-cost and low-power microcontrollers. The system is validated using motion profiles with a vision-based tracking method. The two-link Robot Arm's main goal is to facilitate teaching under the pandemic conditions, supporting laboratory experiments that introduce important robotics topics, such as direct kinematics and inverse kinematics. The mechanical structure is shown in Fig. 1.

## 2 Hardware description.

The robot is a low-cost prototype which is powered by IoT components [1]. With this robotic system, inexperienced students, researchers, and even enthusiasts who do not have access to a robotics laboratory or a diversity of materials and actuators will be able to replicate, comprehend, and verify the resulting behavior motion of a robot built from scratch. Its links are made from PLA filament because of the low amount of material required to print each link and the flexibility and variety in design that a 3D printer allows. Also, because the amount of material used is minimal, the servo motors can lift the weight of the components used in the prototype.

This robot can be controlled by an HMI interface in a mobile phone to move the angle of each servo motor using Denavit - Hartenberg parameters to obtain the direct kinematics matrix and determine its position. Also, the interface can replicate the pattern of motion trajectories using inverse kinematics through geometrical relations between the links via a button that introduces a replication protocol that the robot follows. For purposes of demonstration, we selected the following patterns: a circle, a five-petal flower, and a spiral drawn by hand and processed with an open-source MATLAB code so that the robot could follow its trajectory. The equations of the robot were tested using a MATLAB that simulates the specified dimensions and trajectories the robot would follow in its physical prototype. To make the program accessible as an open-source project and fit the controller with wireless communication, we adapted the MATLAB code to be compatible with Arduino IDE. The program was loaded into ESP32 hardware because of its integrated Wi-Fi and dual-mode Bluetooth.

The researchers, teachers, or students who want to replicate the design would be able to:

• Comprehend the relationship between the equations and the behaviors of the robot.
• Implement their robotic configuration using this prototype as a guide, formulating the equations and executing the physical implementation.
• Be involved in the design of a physical prototype to demonstrate how knowledge is acquired through project-based learning.
• Obtain a low cost, functioning hardware for students to learn coding, electronics, and robotics.
• Use the robot to generate and control motion trajectories via an IoT interface in online experiments that simulate realistic environments.
• Take advantage of a highly customizable system that can be adapted to research and teaching needs.

## 3 Design files

Design Files Summary

**Table:** 

Design file name	File type	Open source license	Location of the file
Robotic Link 1	CAD File	Creative Commons Attribution-ShareAlike 4.0 International License.	https://doi.org/10.17605/OSF.IO/EN9W6
Robotic Link 2	CAD File	Creative Commons Attribution-ShareAlike 4.0 International License.	https://doi.org/10.17605/OSF.IO/EN9W6
Servo Base	CAD File	Creative Commons Attribution-ShareAlike 4.0 International License.	https://doi.org/10.17605/OSF.IO/EN9W6
TOWER PRO MG-995, ANALOG SERVO	CAD File	Creative Commons Attribution-ShareAlike 4.0 International License.	https://grabcad.com/library/tzt-mg-996r-servo-motor-1
Sharpie Fine Point	CAD File	Creative Commons Attribution-ShareAlike 4.0 International License.	https://grabcad.com/library/sharpie-w-fine-point-1
BATTERY_HOLDER	CAD File	Creative Commons Attribution-ShareAlike 4.0 International License.	https://grabcad.com/library/battery-holder-aa-1-5v-1
ESP32S Dev Board 0.9 in. width ASSY	CAD File	Creative Commons Attribution-ShareAlike 4.0 International License.	https://grabcad.com/library/esp-32s-development-board-1
proto_400	CAD File	Creative Commons Attribution-ShareAlike 4.0 International License.	https://grabcad.com/library/protoboard-400-pts-1
Assem1	CAD File	Creative Commons Attribution-ShareAlike 4.0 International License.	https://doi.org/10.17605/OSF.IO/EN9W6
STL Base	STL File	Creative Commons Attribution-ShareAlike 4.0 International License.	https://doi.org/10.17605/OSF.IO/EN9W6
STL Link 1	STL File	Creative Commons Attribution-ShareAlike 4.0 International License.	https://doi.org/10.17605/OSF.IO/EN9W6
STL Link 2	STL File	Creative Commons Attribution-ShareAlike 4.0 International License.	https://doi.org/10.17605/OSF.IO/EN9W6
RobotArd	Arduino File	Creative Commons Attribution-ShareAlike 4.0 International License.	https://doi.org/10.17605/OSF.IO/EN9W6
test1	PNG File	Creative Commons Attribution-ShareAlike 4.0 International License.	https://doi.org/10.17605/OSF.IO/EN9W6
test2	DAT File	Creative Commons Attribution-ShareAlike 4.0 International License.	https://doi.org/10.17605/OSF.IO/EN9W6
digitize2	MATLAB File	Creative Commons Attribution-ShareAlike 4.0 International License.	https://la.mathworks.com/matlabcentral/fileexchange/928-digitize2-m or available at https://doi.org/10.17605/OSF.IO/EN9W6
RobotFinalSIM	MATLAB File	Creative Commons Attribution-ShareAlike 4.0 International License.	https://doi.org/10.17605/OSF.IO/EN9W6

● **Robotic Link 1.sldprt -** SolidWorks part file of the first linkage in the robot. Consists of a hollow body with a slot for another servo and a circular servo horn.
● **Robotic Link 2.sldprt -** SolidWorks part file of the second linkage in the robot. Consists of a hollow body with a slot for a marker pen and a circular servo horn.
● **Servo Base.sldprt -** SolidWorks part file of the base of the robot that has a slot for the first servomotor and the 400-hole protoboard.
● **TOWER PRO MG-995, ANALOG SERVO.sldasm -** A SolidWorks assembly file found in grabcad.com, representing an MG-995 servo.
● **Sharpie Fine Point.sldprt -** A SolidWorks part file found in grabcad.com, representing a marker pen.
● **BATTERY_HOLDER.step -** A SolidWorks part file found in grabcad.com, representing a 4-AA battery pack.
● **ESP32S Dev Board 0.9 in. width ASSY.sldprt -** A SolidWorks part file found in grabcad.com, representing an ESP32 development board microcontroller.
● **proto_400.step -** A SolidWorks part file found in grabcad.com, representing a small protoboard.
● **Assem1.sldasm -** A SolidWorks assembly file of the fully assembled two-link robot.
● **STL Base.stl -** A STL File of the robot's base for 3D printing.
● **STL Link 1.stl -** A STL File of the robot's first arm for 3D printing.
● **STL Link 2.stl -** A STL File of the robot's second arm for 3d printing.
● **RobotArd.ino -** Arduino code that is run in the Esp32.
● **test1.png** - Image of a scanned handwritten spiral to test the image processing.
● **test2.dat** - Generic data file to replicate the image processing test.
● **digitize2.m** - MATLAB file by Anil Prasad (2001) to digitize points based on an image.
● **RobotFinalSIM.m** - MATLAB file that uses Robotics Toolbox 9th Edition by Peter Corke (2015) to simulate the robot behavior.

## 4 Bill of materials

**Table:** 

Component	Number	Cost per unit -currency	Total cost - currency	Source of materials	Material type
Tower Pro MG-995 Servo	2 pcs	$4.99 USD	$9.98 USD	https://www.amazon.com/-/es/KAILEDI-Motors-Walking-Control-Arduino/dp/B083DZ88R6/ref=sr_1_2?__mk_es_US=%C3%85M%C3%85%C5%BD%C3%95%C3%91&crid=1WFS54RUM9TCG&dchild=1&keywords=servo+motor+mg995&qid=1591495062&sprefix=servo+motors+mg99%2Caps%2C210&sr=8–2	Electronic
PLA 3D Printing Filament	4.163 g	$24.99 USD	$1.034 USD	https://www.amazon.com/-/es/SUNLU-Filament-Printer-Printers-LightGold/dp/B07X54WLHX/ref=sr_1_12?__mk_es_US=%C3%85M%C3%85%C5%BD%C3%95%C3%91&crid=JW4POYB4QP3Y&dchild=1&keywords=pla+filament&qid=1591588087&sprefix=pla+%2Caps%2C217&sr=8–12	Polymer
ESP32 Microcontroller	1 pc	$10.99 USD	$10.99 USD	https://www.amazon.com/HiLetgo-ESP-WROOM-32-Development-Microcontroller-Integrated/dp/B0718T232Z/ref=sr_1_1_sspa?__mk_es_US=%C3%85M%C3%85%C5%BD%C3%95%C3%91&dchild=1&keywords=esp32&qid=1591495666&sr=8–1-spons&psc=1&spLa=ZW5jcnlwdGVkUXVhbGlmaWVyPUExUE1KN1Y0SU1TSU5HJmVuY3J5cHRlZElkPUExMDM1NzMxV1VWV1ZZREc5TklVJmVuY3J5cHRlZEFkSWQ9QTAyMDg0MzEyTzNRU1ZWVFJUQTFOJndpZGdldE5hbWU9c3BfYXRmJmFjdGlvbj1jbGlja1JlZGlyZWN0JmRvTm90TG9nQ2xpY2s9dHJ1ZQ==	Electronic
Battery pack 4 AA	1 pc	$5.99 USD	$5.99 USD	https://www.amazon.com/Velleman-BH343B-Battery-AA-Cell-Terminals/dp/B00A41MZ62/ref=sr_1_37?dchild=1&keywords=4+aa+battery+pack&qid=1591565808&sr=8–37	Electronic
Jumper wires	10 pcs	$5.79 USD	$0.4825 USD	https://www.amazon.com/-/es/EDGELEC-Breadboard-Optional-Assorted-Multicolored/dp/B07GD2BWPY/ref=sr_1_3?__mk_es_US=%C3%85M%C3%85%C5%BD%C3%95%C3%91&dchild=1&keywords=jump+wires&qid=1591588133&sr=8–3	Electronic

## 5 Build instructions

**Fig. 2:**
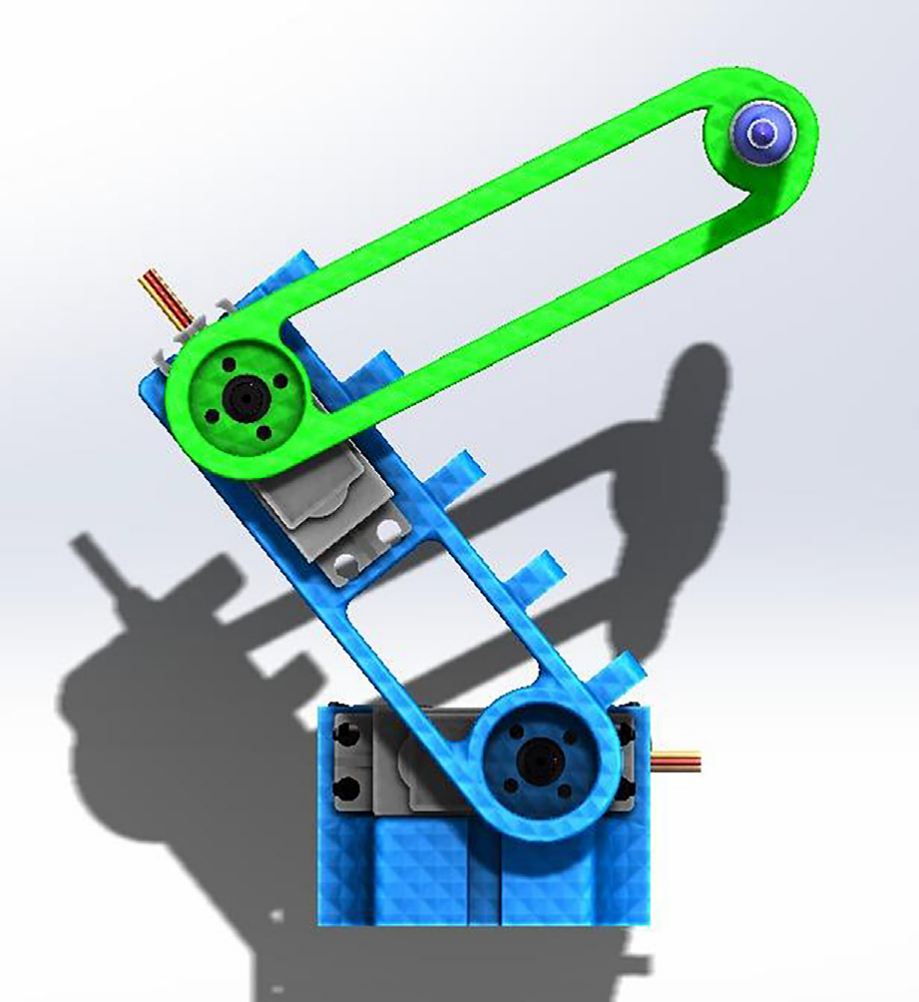
Front view of the robot, showing how the arms are installed.

**Fig. 3:**
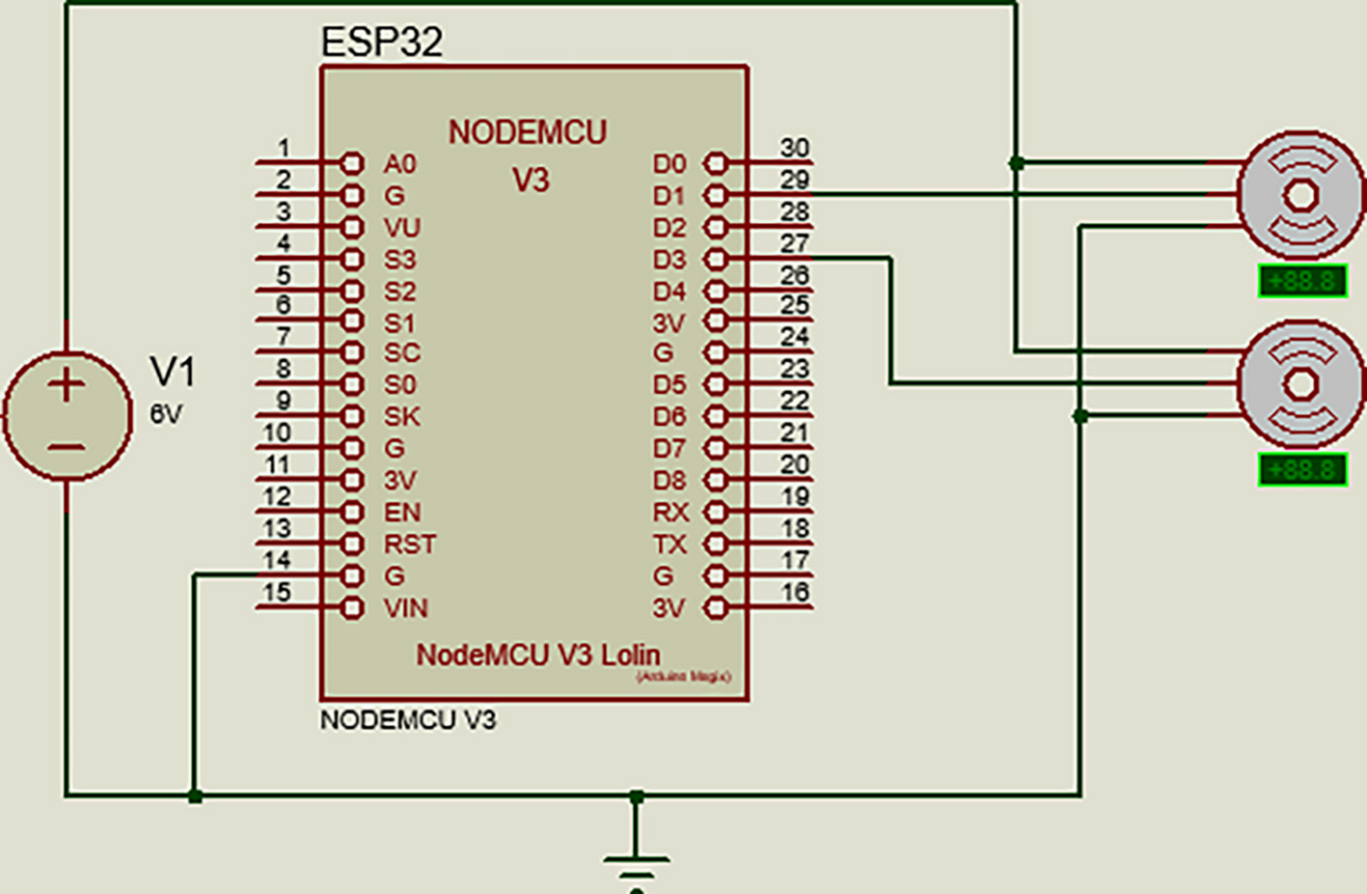
Electrical diagram of the robot.

The first step in building the robot is to acquire and print in 3D all the parts mentioned in the Bill of Materials and the design file summary. Once the parts are ready, the steps are the following:

• Insert the circular servo horns (included with the MG-995 servo) into the two robotic links and secure them with small screws.
• Secure the first servo motor into the servo base and the second servo motor into the first link slot with screws and nuts (6/32 in. screws work well).
• Secure the first robot link into the servo at the base by the circular horn and the second robot link at the second servo motor.
• Place the protoboard at the base of the robot and secure the ESP32.
• Connect the battery pack to the protoboard and connect the grounds of the servo and the battery pack.
• Finally, connect the first and second servo motors to ground, power, and the first and third pins of the microcontroller (Figure 2 and Figure 3).

### 5.1 Direct kinematics

The first step necessary to program the robot is to define its initial position and the workspace available with the servo motors. For convenience, this prototype assumes that both servos are at 0° as the following image shows, where S0 and S1 are the two locations of the servo motors, and S2 is the position at the end of the second link. Also, a1 and a2 are the lengths of each link, respectively.

It is also recommended to build a parameter table to visualize the previous configuration in a sectioned manner and comprehend the relationships between the actuators' behaviors.

● qn: Angle input to the servo motor.
● θn: Desired angle for the robot.
● an: Length of arm or link.
● dn: Offset between links (in this case, both are " because the robot is planar).
● ⍺n: Twist (used when a Z-axis is not aligned with the others in the D-H configuration).

Once the configuration is defined, the direct kinematics matrix can be obtained. Its general structure is as follows:

(1) $$\begin{array}{c} T_{n}=\left[\begin{array}{cccc} \cos\left(\theta n\right) & -\sin\left(\theta n\right)\cos\left(\alpha n\right) & \sin\left(\theta n\right)\sin\left(\alpha n\right) & ancos\left(\theta n\right)\\ \sin\left(\theta n\right) & \cos\left(\theta n\right)\cos\left(\alpha n\right) & -\cos\left(\theta n\right)\sin\left(\alpha n\right) & \mathrm{an}\sin\left(\theta n\right)\\ 0 & \sin\left(\alpha n\right) & \cos(\alpha n) & dn\\ 0 & 0 & 0 & 1 \end{array}\right] \end{array}$$

So, by taking into consideration the values from Table 1 and Eq. (1), a matrix for each link is obtained.

(2) $$\begin{array}{c} T_{1}=\left[\begin{array}{cccc} \cos\left(\theta 1\right) & -\sin\left(\theta 1\right) & 0 & 84.8\cos\left(\theta 1\right)\\ \sin\left(\theta 1\right) & \cos\left(\theta 1\right) & 0 & 84.8\sin\left(\theta 1\right)\\ 0 & 0 & 1 & 0\\ 0 & 0 & 0 & 1 \end{array}\right] \end{array}$$

(3) $$\begin{array}{c} T_{2}=\left[\begin{array}{cccc} \cos\left(\theta 2\right) & -\sin\left(\theta 2\right) & 0 & 106\cos\left(\theta 2\right)\\ \sin\left(\theta 2\right) & \cos\left(\theta 2\right) & 0 & 106\sin\left(\theta 2\right)\\ 0 & 0 & 1 & 0\\ 0 & 0 & 0 & 1 \end{array}\right] \end{array}$$

**Table 1:** D-H parameters from the chosen configuration.

Link	qn	an	dn	⍺n
S1	q1 = θ1	a1 = 84.8 mm	d1 = 0	⍺1 = 0
S2	q2 = θ2	a2 = 106 mm	d2 = 0	⍺2 = 0

Multiplying (2), (3) the following general direct kinematics matrix is obtained:

(4) $$\begin{array}{c} T=\left[\begin{array}{cccc} \cos\left(\theta 1+\theta 2\right) & -\sin\left(\theta 1+\theta 2\right) & 0 & 84.8\cos\left(\theta 1\right)+106\cos\left(\theta 1+\theta 2\right)\\ \sin\left(\theta 1+\theta 2\right) & \cos\left(\theta 1+\theta 2\right) & 0 & 84.8\sin\left(\theta 1\right)+106\sin\left(\theta 1+\theta 2\right)\\ 0 & 0 & 1 & 0\\ 0 & 0 & 0 & 1 \end{array}\right] \end{array}$$

In the home position, the matrix (4) is evaluated numerically as

$$\mathrm{where}\;\theta_{1}=0^{{}^{\circ}}\;and\;\theta_{2}=0^{{}^{\circ}}\to T=\left[\begin{array}{cccc} 1 & 0 & 0 & 190.8\\ 0 & 1 & 0 & 0\\ 0 & 0 & 1 & 0\\ 0 & 0 & 0 & 1 \end{array}\right]$$

**Fig. 4:**
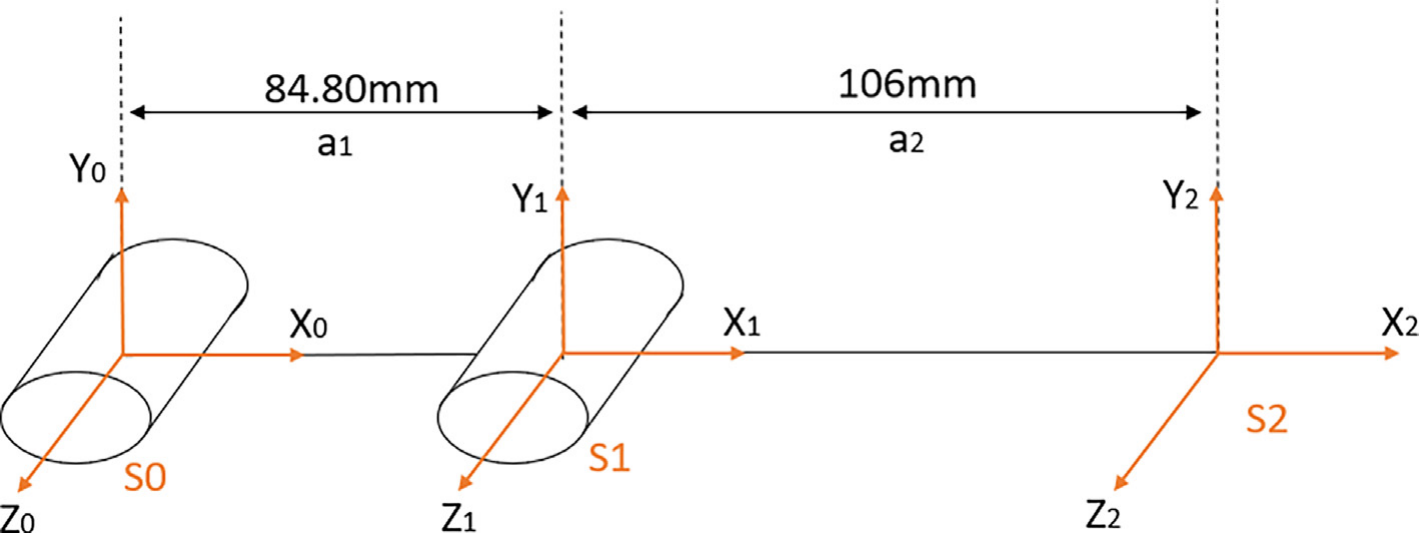
Denavit-Hartenberg configuration for the robot in the HOME position.

In the previous matrix, the first three columns and rows correspond to the current axis of the final effector in relationship to the global axis of the system defined in the DH configuration shown in Fig. 4. For example, the number in the first row and column is 1, and so it indicates that the X-axis is in line with its original intended direction at 0° in both servo motors. The final column corresponds to the actual coordinate of the final effector. In the previous configuration, the effector is at (190.8, 0), or 190.8 mm to the right from the position (0, 0).

### 5.2 Inverse kinematics

**Fig. 5:**
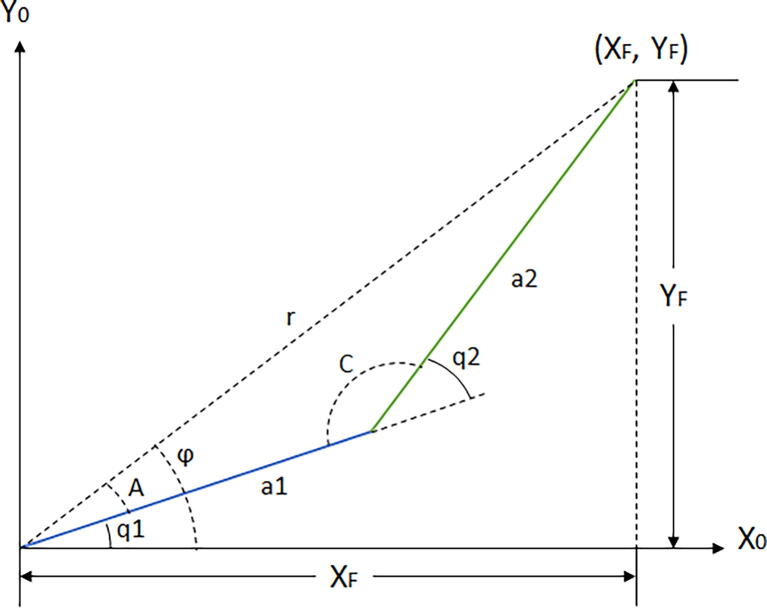
Proposed robot position for inverse kinematic analysis.

To implement inverse kinematics by geometric relations correctly, one must be sure that the equations of the robot have all the necessary elements to find the desired angle, given a set of coordinates. There are multiple solutions to a single instruction, depending on how the equations are solved. To make a solution proposal, one must first put the robot in a position where all its angles are visible. The HOME position cannot be used because, in that position, the angles are not visible. Hence, the proposal is to begin with the inverse kinematics solution of this robot and to move to the second link, as depicted in Fig. 5:

where:

● a1 and a2 are the lengths of each link.
● q1 and q2 are the desired angles the robot must determine.
● XF, YF, r, and φ are auxiliary variables for finding relations between the links.
● A and C are auxiliary variables to find relations between the links using trigonometric relationships.

After defining the model of Fig. 5, the equations of q1 and q2 can be found using trigonometric and algebraic knowledge.

The Fig. 5 shows that it is easy to verify that:

(5) $$\begin{array}{c} r^{2}={X_{F}}^{2}-{Y_{F}}^{2} \end{array}$$

Proposal to find q1:

(6) $$\begin{array}{c} q_{1}=\varphi -A \end{array}$$

(7) $$\begin{array}{c} \varphi =\tan^{-1}\left(\frac{Y_{F}}{X_{F}}\right) \end{array}$$

Applying the Law of Cosines for auxiliary angle A:

(8) $$\begin{array}{c} a_{2}^{2}=r^{2}+a_{1}^{2}-2ra_{1}\cos\left(A\right) \end{array}$$

(9) $$\begin{array}{c} \cos\left(A\right)=\frac{r^{2}+a_{1}^{2}-a_{2}^{2}}{2ra_{1}} \end{array}$$

Using trigonometric identity to find the sine of auxiliary angle A:

(10) $$\begin{array}{c} \sin\left(A\right)=\sqrt{1-{\left(\frac{r^{2}+a_{1}^{2}-a_{2}^{2}}{2ra_{1}}\right)}^{2}} \end{array}$$

Eqs. (9), (10) are used to find the tangent of auxiliary angle A:

(11) $$\begin{array}{c} A=\tan^{-1}\left(\frac{\sqrt{1-{\left(\frac{r^{2}+a_{1}^{2}-a_{2}^{2}}{2ra_{1}}\right)}^{2}}}{\frac{r^{2}+a_{1}^{2}-a_{2}^{2}}{2ra_{1}}}\right) \end{array}$$

By substituting (7), (11) into Eq. (6), it is possible to find the generalized coordinate q1 as:

(12) $$\begin{array}{c} q_{1}=\tan^{-1}\left(\frac{Y_{F}}{X_{F}}\right)-\tan^{-1}\left(\frac{\sqrt{1-{\left(\frac{r^{2}+a_{1}^{2}-a_{2}^{2}}{2ra_{1}}\right)}^{2}}}{\frac{r^{2}+a_{1}^{2}-a_{2}^{2}}{2ra_{1}}}\right) \end{array}$$

From Fig. 5, it is possible to find q2:

(13) $$\begin{array}{c} q_{2}=\pi -C \end{array}$$

The Law of Cosines for auxiliary angle C:

(14) $$\begin{array}{c} r^{2}=a_{1}^{2}+a_{2}^{2}-2a_{1}a_{2}\cos\left(C\right) \end{array}$$

(15) $$\begin{array}{c} \cos\left(C\right)=\frac{a_{1}^{2}+a_{2}^{2}-r^{2}}{2a_{1}a_{2}} \end{array}$$

Using trigonometric identity to find the sine of auxiliary angle C:

(16) $$\begin{array}{c} \sin\left(C\right)=\sqrt{1-{\left(\frac{a_{1}^{2}+a_{2}^{2}-r^{2}}{2a_{1}a_{2}}\right)}^{2}} \end{array}$$

Eqs. (15), (16) are used to find the tangent of auxiliary angle C:

(17) $$\begin{array}{c} C=\tan^{-1}\left(\frac{\sqrt{1-{\left(\frac{a_{1}^{2}+a_{2}^{2}-r^{2}}{2a_{1}a_{2}}\right)}^{2}}}{\frac{a_{1}^{2}+a_{2}^{2}-r^{2}}{2a_{1}a_{2}}}\right) \end{array}$$

By substituting (17) into (13):

(18) $$\begin{array}{c} q_{2}=\pi -\tan^{-1}\left(\frac{\sqrt{1-{\left(\frac{a_{1}^{2}+a_{2}^{2}-r^{2}}{2a_{1}a_{2}}\right)}^{2}}}{\frac{a_{1}^{2}+a_{2}^{2}-r^{2}}{2a_{1}a_{2}}}\right) \end{array}$$

### 5.3 Simulations

The equations obtained above can be implemented directly in Arduino. However, to ensure that the robot behaves adequately, it is recommended that inverse kinematic Eqs. (12), (18) are tested in simulation. A simulation code in MATLAB based on [2] was designed using Robotics Toolbox 9th Edition by Peter Corke (2015). The simulation is an additional step that safely tests the robot while implementing different parametric equations of determined shapes and observing that the obtained proposal for angles q1 and q2 works as intended. Because the simulation can replicate multiple shapes, the user must comment on all parametric equations except the one that wants to be tested. The MATLAB file to test this robot is included in the Source File Repository.

**Fig. 6:**
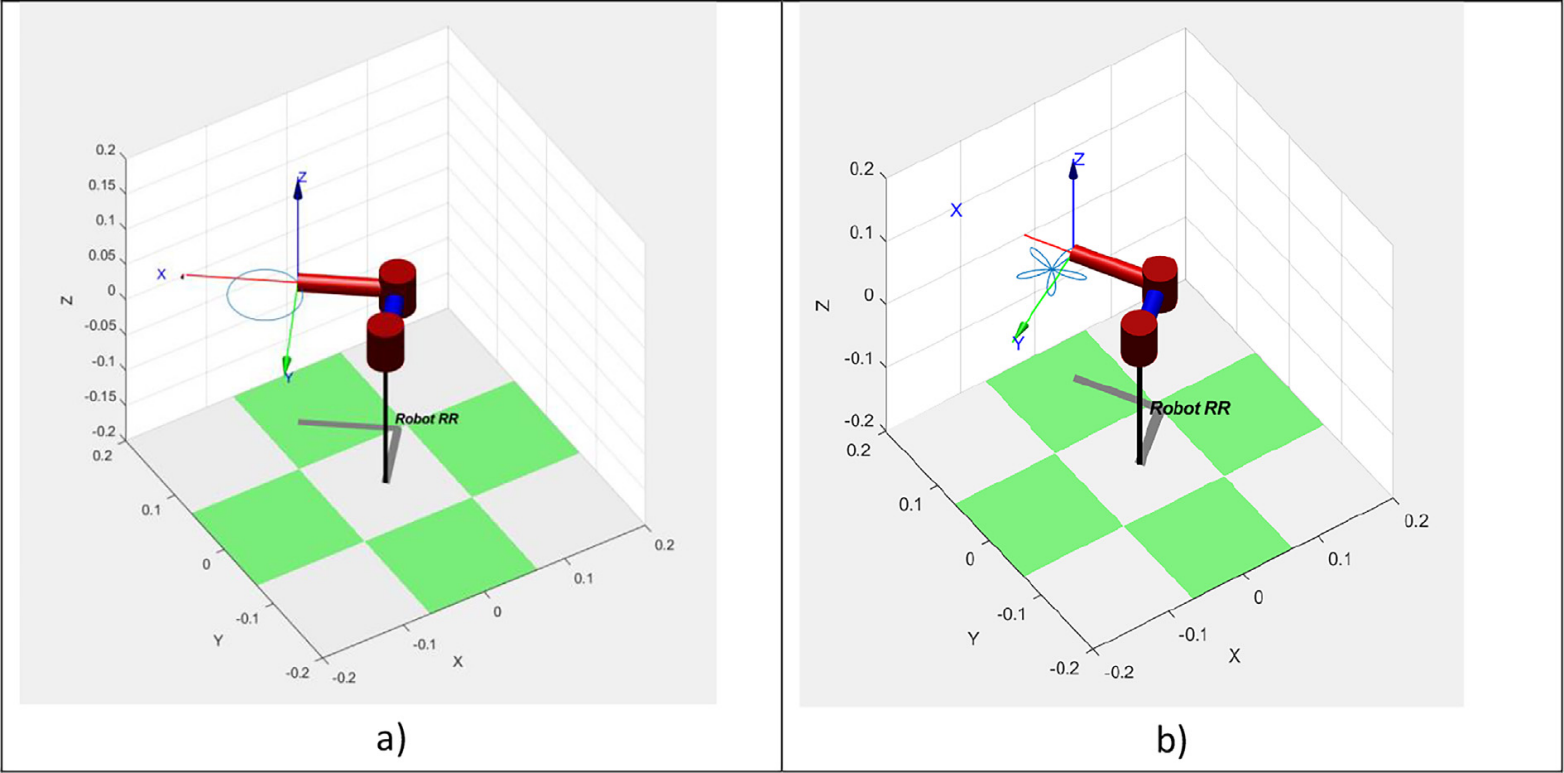
Simulation of the robot replicating a circle, shown in subfigure a), and the five-petal flower, depicted in subfigure b), from parametric equations of inverse kinematics.

**Fig. 7:**
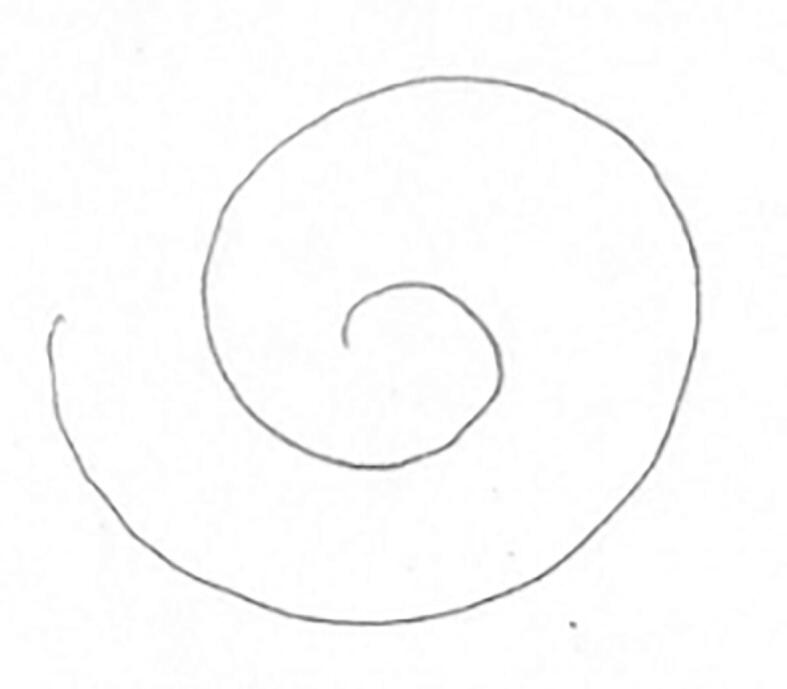
Handwritten spiral scanned to be replicated by the robot.

**Fig. 8:**
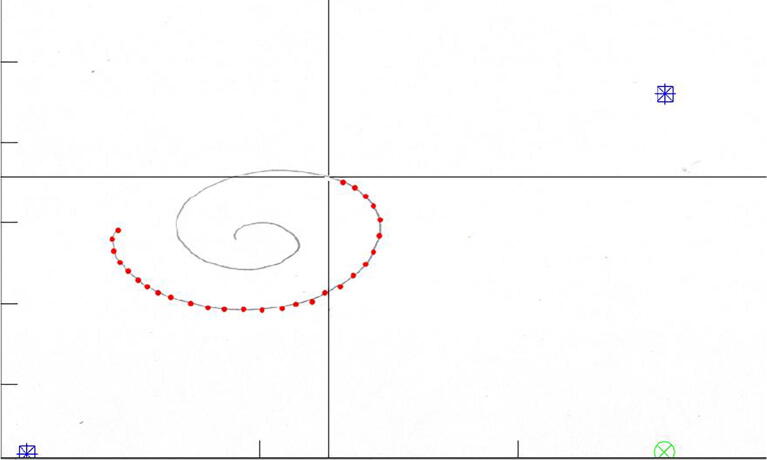
Example of the process of point selection from an image.

As Fig. 6a and Fig. 6b demonstrate, the robot indeed replicates the desired figures with the proposed solution obtained in (12), (18). To further test the capabilities of the robot, we implemented a way to replicate any custom figure input by the user. This method uses an open-source MATLAB program uploaded to the MATLAB Central File exchange [3]. It is a code that allows the user to digitalize a set of points from an image. For this test, a spiral was scribbled on a paper with no guidelines or symmetry, as shown in Fig. 7 and its processing output in Fig. 8.

**Fig. 9:**
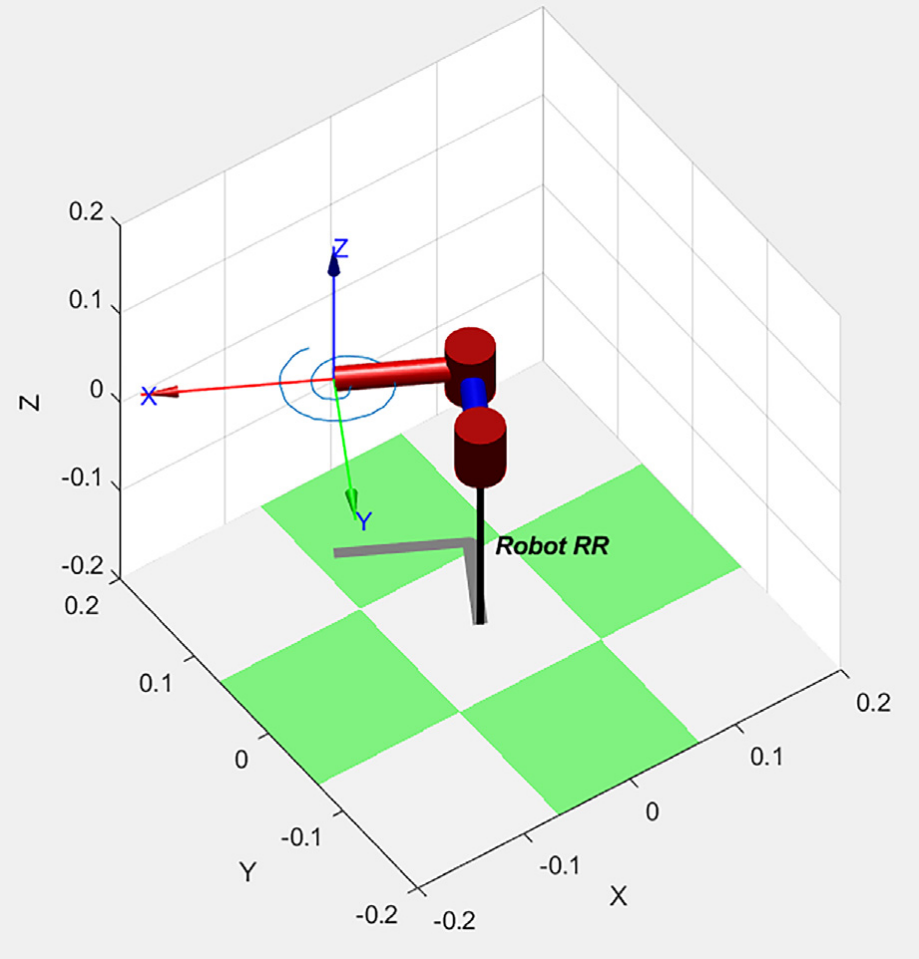
Simulation of the robot replicating the handwritten spiral.

The simulation output custom made spiral is depicted in Fig. 9.

### 5.4 IoT interface

To empower the capabilities of the robot, we specified that the robot should be capable of working online and being controlled by a smartphone. To achieve this, we decided to use the Blynk application [4]. Blynk is a smartphone app with complete documentation and easy-to-understand functions, allowing the user to create a server and a connection between a microcontroller and a smartphone via Arduino for free, as well as a completely customizable interface for each project. The documentation on the Blynk website allowed us to develop the interface and the connection with the microcontroller in a matter of hours; the only remaining problem was to translate the MATLAB code to Arduino.

**Fig. 10:**
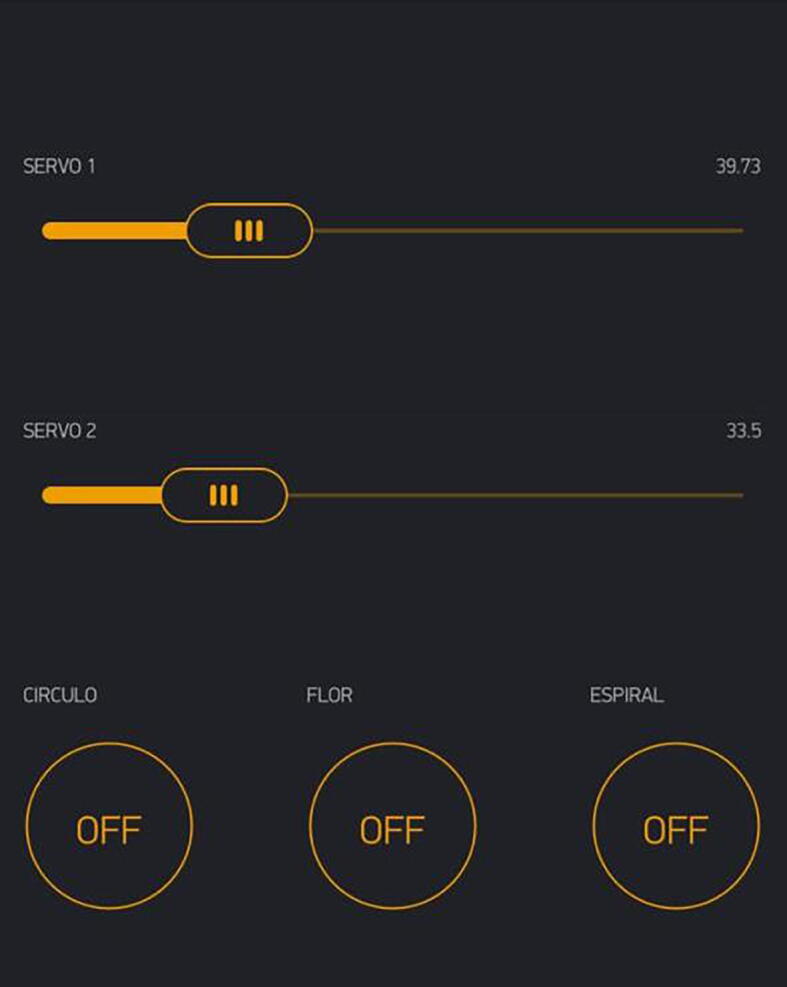
Blynk smartphone app that is used to control the robot.

The following was achieved with the math.h library in Arduino that adds some important functions for inverse kinematics, such as atan2(). The equations themselves, both being written in c, are similar in each program, making their translation an easy step. Finally, for each parametric equation or shape desired, a function was implemented that enclosed all the code needed for that specific movement, as well as the coordinates arrays used for the spiral shown above. The interface with the kinematic implementation is shown in the Fig. 10.

## 6 Operation instructions

● After adjusting the authentication token and the Wi-Fi connection in the Arduino code, connect the microcontroller to the computer via USB and upload the code.
● While uploading code to the microcontroller, make sure that the power supply (6 V battery pack) for the servos is disconnected. While uploading, the pins on the ESP32 might send random signals and cause the servos to move if the battery is still connected.
● As soon as the code is uploaded or the coded ESP32 is connected for the first time, the ESP32 will attempt to connect to the internet.
● If the Wi-Fi signal is strong enough, the microcontroller will appear available in the Blynk app.
● From the phone, control the angle of each servo individually from 0 to 180°with the sliders and activate the pre-programmed functions with the buttons.
● Before changing the values of the rotation, the parametric functions, or the set of coordinates in the robot's code, make sure that the values you are attempting are real values that the robot can fulfill. When using wrong values, the servos can make abrupt movements that may damage the robot and their surroundings.

## 7 Validation and characterization

**Fig. 11:**
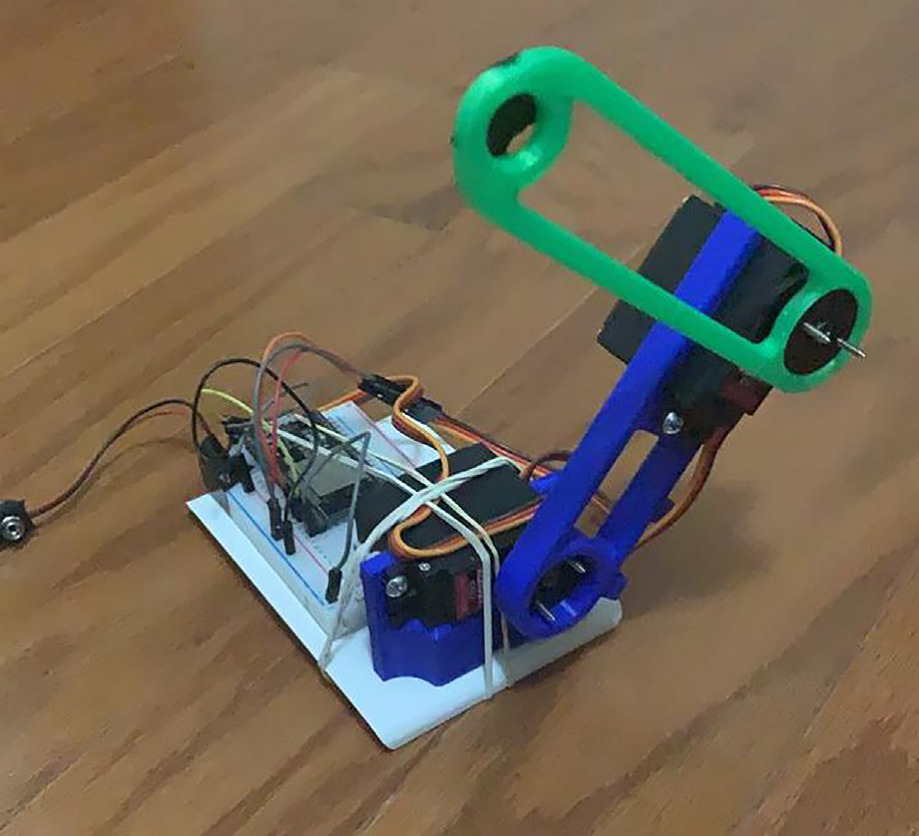
Robot prototype (Front view).

**Fig. 12:**
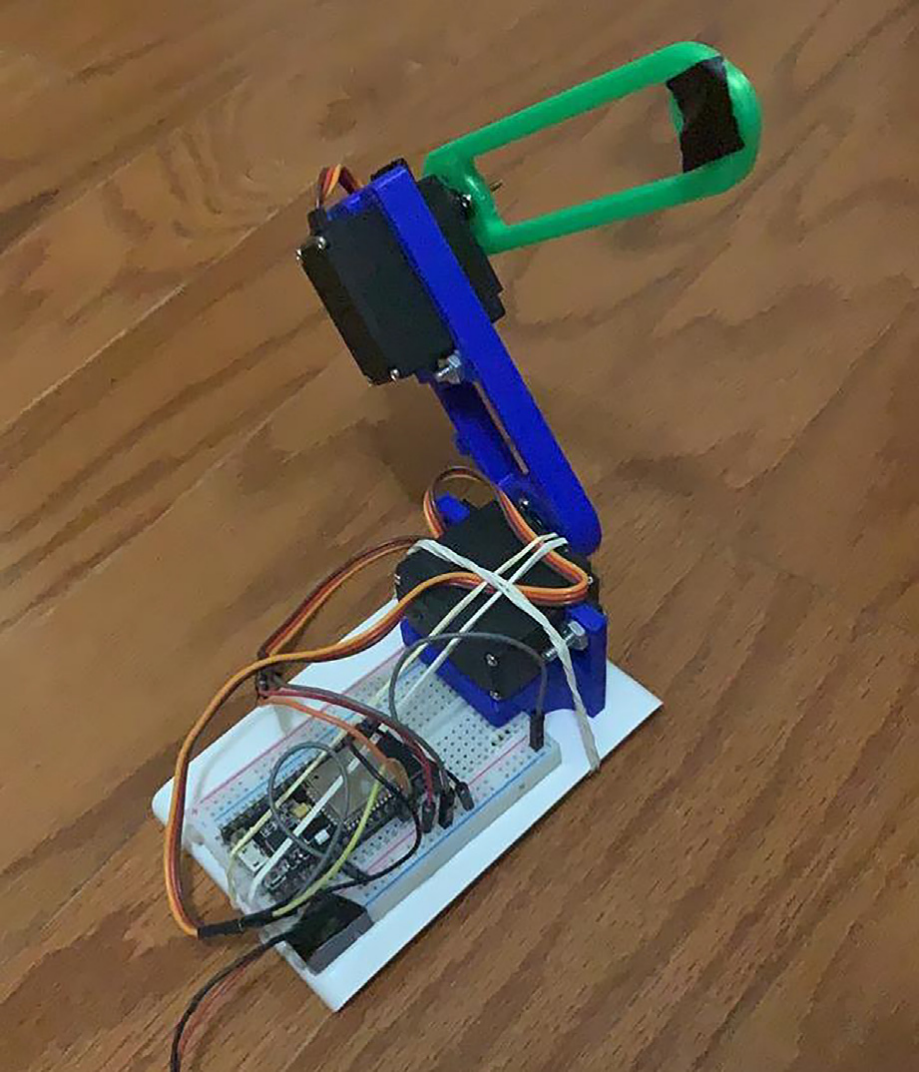
Robot prototype (Back view).

To demonstrate the operation and performance of the hardware, we built a prototype using the components described in the Bill of Materials (Fig. 11, Fig. 12).

To evaluate the motion control of the prototype, we made a video of the robot replicating the patterns in Fig. 6, Fig. 9 and loaded it into Tracker. Tracker is a free video-analysis-and-modeling tool built on the Open Source Physics (OSP) Java framework [5]. When a reference point to be followed is defined, the program can track the pattern that follows the established reference frame by frame. It compares the position and color of the pixels defined as references. The results of the physical prototype obtained when analyzing the trajectories were plotted automatically to see the pattern followed in the tests.

Parametric equation used to code the trajectories are:

**Parametric curve used for the circle:**

Fx[i] = -0.07 + 0.04*cos(rad);

Fy[i] = 0.13 + 0.04*sin(rad);

**Parametric curve used for the flower:**

Fx[i] = -0.06 + 0.04*(1 + sin(5*rad)*cos(rad));

Fy[i] = 0.1 + 0.04*(1 + sin(5*rad)*sin(rad));

**Parametric curve used for the spiral:**

As it was done using digitize library for MATLAB, the coordinates for the spiral are obtained as a data table, meaning that our program goes throughout different saved points plotting them in a discrete sequence.

**Fig. 13:**
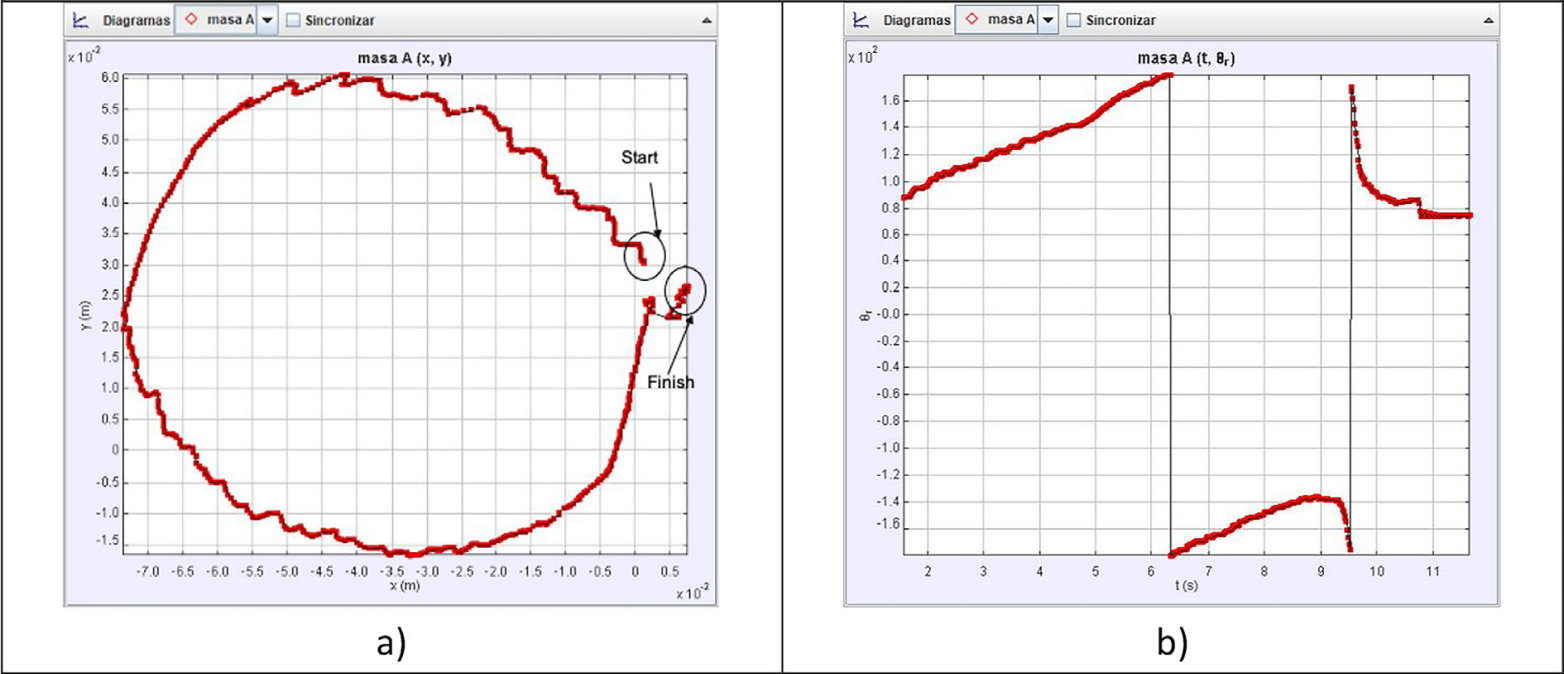
Circle pattern tracked by the physical prototype, shown in subfigure a), and the angle vs time, depicted in subfigure b).

**Fig. 14:**
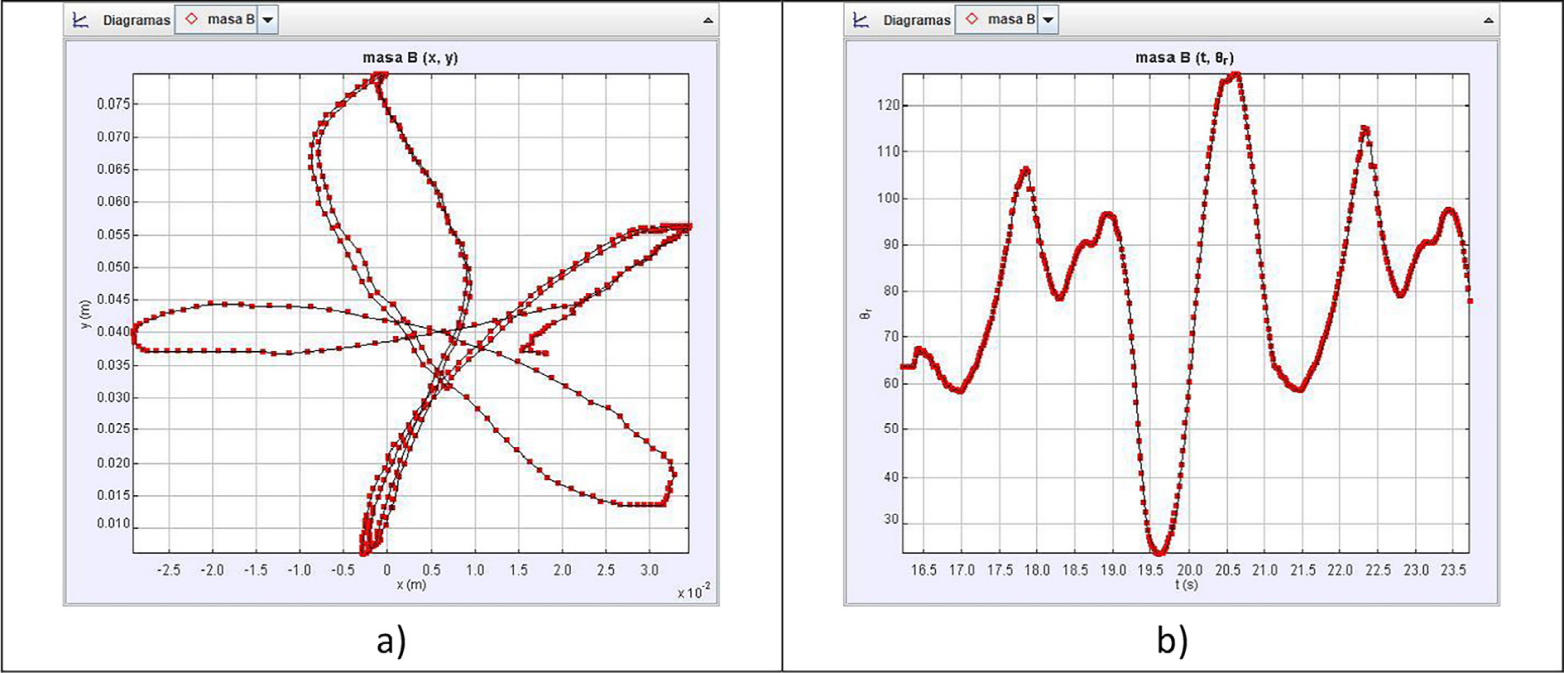
Five-petal flower pattern tracked by the physical prototype, shown in subfigure a), and the angle vs time, depicted in subfigure b).

**Fig. 15:**
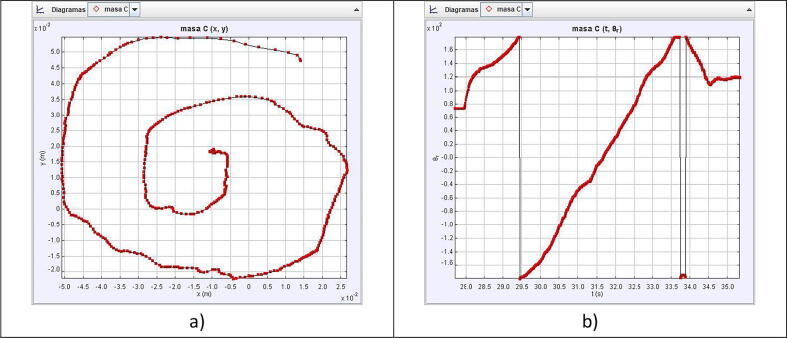
Handwritten spiral pattern tracked by the physical prototype, shown in subfigure a), and the angle vs time, depicted in subfigure b).

As Fig. 13, Fig. 14, Fig. 15 show, the robot motion only approximates the real trajectories that the robot must follow. This is due to the following factors:

● The weight of the servos used represent extra effort not considered in Eqs. (12), (18).
● The quality of the servo motors is not optimal but is affordable for the demonstration.
● The dynamic model of the robot was not considered.
● The friction between mechanical components.
● Arduino libraries accurate issues [6].
● Information is lost when transferring information to the servo motors using the PWM signal.

Nonetheless, the results obtained by the prototype are acceptable and adequately demonstrate the correct design and operation of a robot arm. A video clip is provided in the Source File Repository, where the motion tracking of patterns is shown.

## Declaration of Competing Interest

The authors declare that they have no known competing financial interests or personal relationships that could have appeared to influence the work reported in this paper.
